# Laminitis in Holstein dairy cows is associated with intestinal and mammary dysfunction, systemic inflammation, and microbiota dysregulation

**DOI:** 10.3389/fmicb.2026.1879424

**Published:** 2026-06-24

**Authors:** Wen Peng, Juan Chen, Xiaona Gao, Xiaoquan Guo, Pei Liu, Gaofeng Cai, Zhanhong Zheng, Gen Wan, Ping Liu

**Affiliations:** College of Animal Science and Technology, Jiangxi Agricultural University, Nanchang, China

**Keywords:** 16S rRNA, fecal microbiota, Holstein dairy cows, laminitis, milk microbiota

## Abstract

Laminitis is a major health and production disorder in dairy cows, yet its systemic associations with host physiology and microbiota remain incompletely understood. This exploratory study investigated the relationships among lameness severity, production performance, serum biomarkers, and milk and fecal microbiota. Cows were classified into healthy and laminitic groups using locomotion scoring. While milk composition remained largely stable, laminitic cows exhibited significantly reduced milk yield. Serum analyses revealed elevated inflammatory cytokines, oxidative stress markers, stress hormones, and intestinal barrier indicators, including lipopolysaccharide, diamine oxidase, and D-lactate. Fecal microbiota structure differed significantly between groups, whereas milk microbiota showed limited overall variation. Correlation and network analyses demonstrated preliminary coordinated associations among lameness, reduced production, systemic inflammation, and barrier dysfunction. Furthermore, PICRUSt-based predictions suggested an enriched genetic potential for central carbon metabolism and transcription-related pathways in laminitic cows, while healthy cows showed higher predicted capacities for nutrient processing. Collectively, these exploratory findings provide preliminary evidence that laminitis is associated with systemic physiological disruption and altered predicted microbial capacities. We propose a potential microbiota-associated gut–systemic inflammatory link, accompanied by secondary impairments in mammary physiological function, warranting future validation in larger-scale longitudinal cohorts.

## 1 Introduction

Laminitis, traditionally characterized as diffuse aseptic pododermatitis, is one of the most debilitating metabolic disorders in modern high-yield dairy farming, inflicting severe economic burdens on the global dairy industry (Hayat et al., 2025). Although its pathogenesis is not fully understood, it has been widely proposed that laminitis may be associated with dietary and metabolic disturbances, particularly those related to high-concentrate feeding. Historically, it has been proposed that such dietary conditions might promote subacute ruminal acidosis (SARA), which has been hypothesized in literature to lead to the release of pro-inflammatory compounds, including lipopolysaccharides (LPS) and histamine (Passos et al., 2023). Upon entering the systemic circulation, these endotoxins accumulate in the hoof corium, triggering severe microcirculatory dysfunction and tissue edema. This process is further exacerbated by an LPS-induced cascade of pro-inflammatory cytokines and metalloproteinases, which fundamentally degrades the local lamellar tissue (Bergsten, 2003; Nocek, 1997). While this SARA-endotoxemia model provides one possible theoretical framework, the exact mechanistic pathways—whether driven primarily by ruminal disturbances, alternative gastrointestinal dysfunctions, or other systemic stressors—remain a subject of ongoing investigation.

As the most ancient and ubiquitous organisms on Earth, microorganisms constitute an indispensable component of the biosphere and forge profound symbiotic relationships with their hosts (Yang et al., 2025). However, this host microbiome homeostasis is highly dynamic and susceptible to perturbations from external stressors, such as environmental fluctuations, dietary shifts, and pharmacological interventions (Nagata et al., 2022). The consequent microbial dysbiosis not only disrupts intrapopulation dynamics but also fundamentally impairs host physiological functions, precipitating a cascade of metabolic and immunological disorders (Zhong et al., 2026). In modern dairy farming, the stability of the gut and milk microbiomes is of paramount importance for systemic health. The intestinal microbiota serves as a central metabolic and immunological hub. Its metabolites, notably short chain fatty acids, regulate the proliferation of intestinal epithelial cells and drive the maturation of the mucosal immune system (Yao et al., 2022). Conversely, gastrointestinal dysbiosis is intricately linked to the pathogenesis of various conditions, including digestive dysfunction, metabolic imbalances, and mastitis (Veneman et al., 2015; Wang et al., 2016). Similarly, the milk microbiome acts as a sensitive monitor of the local microenvironment and immune status of the mammary gland. Commensal taxa, such as Lactobacillus and Bifidobacterium, orchestrate localized immune responses and competitively exclude opportunistic pathogens like *Staphylococcus aureus* through nutrient competition and the secretion of antimicrobial metabolites (Lirot et al., 2025; Wang X. et al., 2026). However, whether laminitis is accompanied by coordinated alterations in gut and mammary microbiota remains unclear.

Although laminitis is primarily considered a localized hoof disorder, it may have broader physiological consequences beyond the locomotor system. In dairy cows, laminitis is often accompanied by altered feeding behavior, pain-induced stress, and systemic inflammation, which may secondarily disrupt intestinal homeostasis and mammary physiological function. While the gut plays a central role in barrier integrity and systemic immune modulation, whether laminitis is correlated with concurrent gut microbiota dysbiosis and systemic physiological imbalance remains incompletely characterized. Therefore, this exploratory study aimed to investigate the systemic implications of laminitis by integrating production performance, serum biomarkers (including inflammatory and intestinal barrier indicators), and 16S rRNA microbiota profiles from fecal and milk samples. By evaluating these multi-level parameters, we sought to elucidate whether laminitis is associated with a gut–systemic inflammatory link, accompanied by secondary impairments in mammary productivity.

## 2 Materials and methods

### 2.1 Clinical assessment of laminitis severity

Laminitis scoring was conducted using a standardized clinical evaluation system adapted from established methods (Sprecher et al., 1997; Cook, 2003). All cows were examined by trained veterinarians who were completely blinded to the animals’ prior health and production records. Animals were scored on a 4-point scale (0–3) based on gait, posture, weight-bearing behavior, hoof sensitivity, and overall mobility. Scores were defined as follows: 0, normal locomotion; 1, mild laminitis with slight gait abnormalities or stiffness; 2, moderate laminitis characterized by obvious lameness, shortened stride, arched back, and increased hoof sensitivity; and 3, severe laminitis with pronounced lameness, reluctance to move, and marked pain response. Assessments were performed under consistent environmental conditions while animals were standing and walking on a flat surface. Each cow was independently scored by two observers, and discrepancies were resolved by consensus. For subsequent analyses, cows were classified as healthy (score = 0) or laminitic (score ≥ 1). Laminitis severity was further stratified into mild (score = 1), moderate (score = 2), and severe (score = 3) categories when applicable. From these pools, specific subjects were then selected to form biologically matched pairs for downstream sampling.

### 2.2 Animals and sample collection

This study was conducted at a commercial dairy farm in Guangxi, China, which maintains a herd of approximately 1,800 Holstein cows. All animals were housed in identical free-stall barns equipped with rubber mats and provided ad libitum access to clean water and a total mixed ration (TMR). The ingredients and nutrient composition of the basal TMR were formulated to meet the nutritional requirements of lactating dairy cows and are detailed in Supplementary Table S1. Clinical characteristics and hoof lesion types of the laminitic dairy cows are detailed in Supplementary Table S2. To minimize confounding factors, strict exclusion criteria were applied: the selected subjects had no history of antimicrobial or anti-inflammatory therapy within the 6 months prior to sample collection, and cows with concurrent metabolic or reproductive disorders (e.g., clinical mastitis, ketosis, or retained placenta) were strictly excluded.

Biological samples, specifically milk and fecal samples, were aseptically collected from clinically diagnosed laminitis-affected cows and healthy controls. The groups were biologically matched for parity (average 2.4 ± 0.6) and days in milk (DIM, average 155 ± 21 days). To eliminate potential environmental and cross-contamination during milk sampling, a rigorous aseptic protocol was employed. The udders and teats were thoroughly washed, dried, and disinfected with 75% ethanol. After discarding the first three squirts of foremilk, milk was collected directly into sterile tubes by operators wearing sterile gloves. Fecal samples were collected aseptically from the rectum. All specimens were immediately transferred into sterile centrifuge tubes, transported to the laboratory under cold chain conditions, and cryopreserved for downstream analysis.

A total of 21 samples were successfully obtained and categorized into four experimental groups: milk from laminitis-affected cows (HM, *n* = 5), fecal samples from laminitis-affected cows (HF, *n* = 5), milk from healthy control cows (NM, *n* = 5), and fecal samples from healthy control cows (NF, *n* = 6). Subsequently, the samples were submitted to a commercial biotechnology facility for 16S rRNA gene library preparation and high-throughput sequencing. During sequencing preparation, DNA extraction blanks and PCR no-template controls were included as negative controls to effectively monitor and filter any potential kit or laboratory background contamination.

### 2.3 Assessment of production performance and serum biomarkers

Milk production performance was assessed based on milk yield and composition. Milk fat (MF), protein (MP), lactose (ML), solids-not-fat (MSNF), total solids (MTS), freezing point (MFP), density (MD), electrical conductivity (EC), and ash content were determined using an automated milk analyzer (MilkoScan, Foss, Denmark). Milk yield (MY), fat yield (MFY), protein yield (MPY), energy-corrected milk (ECM), and 3.5% fat-corrected milk (FCM) were calculated from production records. Dry matter intake (DMI), feed efficiency (FE), and 305-day milk yield (305-d MY) were recorded to evaluate production efficiency. Body condition score (BCS), parity, and days in milk (DIM) were obtained from farm records. Blood samples were collected via jugular venipuncture, and serum was obtained by centrifugation (3,000 × g, 10 min) after clotting at room temperature. Serum was stored at −80 °C until analysis. Serum biochemical parameters, including TP, ALB, GLB, ALB/GLB, ALT, AST, ALP, TBA, γ-GGT, BUN, CRE, LDH, GLU, TC, TG, HDL-C, and LDL-C, were measured using an automated biochemical analyzer (Hitachi 7600, Japan). Energy metabolism indicators (NEFA and BHB), oxidative stress markers (SOD, GSH-Px, T-AOC, T-GSH, CAT, and MDA), and inflammatory and immune factors (IL-1β, IL-6, IL-10, HPT, TNF-α, SAA, IgG, and IgA)(SEKB-0363, SEKB-0365, SEKB-0362, SEKP-0031, SEKB-0303, SEKM-0212, SEKB-0335, SEKB-0002, Solarbio, Beijing, China) were determined using commercial assay kits or ELISA kits following the manufacturers’ instructions. Endotoxin and intestinal barrier-related markers, including LPS, DAO, D-lactate, and MMP-9, as well as histamine and cortisol, were also quantified using commercial kits. All assays were performed in triplicate with appropriate quality controls.

### 2.4 Genomic DNA extraction

Total genomic DNA was extracted from the milk and fecal samples using a commercial DNA extraction kit(SB-D402, share-bio, Shang Hai), strictly following the instructions provided by the manufacturer. The yield and purity of the isolated DNA were quantified utilizing a NanoDrop 2000 spectrophotometer (Thermo Fisher Scientific, Wilmington, DE, USA). Genomic integrity was subsequently verified via 1% agarose gel electrophoresis. All validated DNA aliquots were cryopreserved at minus 80 °C pending downstream library construction and sequencing.

### 2.5 PCR amplification

The V3 to V4 hypervariable regions of the 16S rRNA gene were amplified utilizing specific primers and the isolated genomic DNA as a template. Forward primer 338F (5′-ACTCCTACGGGAGGCAGCAG-3′) carrying a barcode sequence and a reverse primer 806R (5′-GGACTACHVGGGTWTCTAAT-3′). All polymerase chain reaction amplifications were executed in triplicate within a 20 μL reaction volume in 200μL PCR tube(PCR-02, A-GEN). This standardized mixture comprised 4 μL of 5× FastPfu Buffer, 2 μL of 2.5 mM dNTPs, 0.8 μL of each forward and reverse primer at a concentration of 5 μM, 0.4 μL of Polymerase(Cat. No.:E001-03; Noveprotein, Shanghai, China), and 10 ng of template DNA. The thermal cycling profile was initiated with a primary denaturation step at 95 °C for 3 minutes. This was followed by 35 amplification cycles consisting of denaturation at 95 °C for 30 seconds, primer annealing at 55 °C for 30 seconds, and strand extension at 72 °C for 45 seconds. The process concluded with a final extension phase at 72 °C for 10 minutes to ensure complete amplicon synthesis (Jiang et al., 2023).

### 2.6 Library construction and purification

The amplified PCR products were excised from 2% agarose gels and subsequently purified utilizing the AxyPrep DNA Gel Extraction Kit (Axygen Biosciences, Union City, CA, USA). The concentration of the purified amplicons was then precisely quantified utilizing the QuantiFluor ST fluorometer system (Promega, Madison, WI, USA). To prepare for high throughput sequencing, these purified amplicons were pooled in equimolar ratios. Finally, paired end sequencing libraries were generated utilizing the NEXTFLEX Rapid DNA Seq Kit (Bioo Scientific, Austin, TX, USA), strictly adhering to the standardized protocols provided by the manufacturer (Jiang et al., 2023).

### 2.7 High-throughput sequencing

High throughput sequencing of the finalized libraries was performed utilizing an Illumina NovaSeq 6,000 platform (Illumina, San Diego, CA, USA). All sequencing procedures strictly adhered to the standardized protocols formulated by Novogene Technology Co., Ltd. (Beijing, China).

### 2.8 Sequencing processing and taxonomic annotation

Raw paired-end reads were merged using FLASH v1.2.11. Quality filtering and demultiplexing were performed using QIIME (v1.9.1) to obtain high-quality sequences. Following quality control, these refined sequences were clustered into operational taxonomic units (OTUs) based on a 97 percent sequence similarity threshold utilizing the UPARSE pipeline. For taxonomic annotation, representative sequences from each OTU were analyzed utilizing the Mothur algorithm against the SSU Ref database, applying a strict confidence threshold ranging from 0.8 to 1.0 (Table 1). This procedure facilitated the comprehensive profiling of species composition across all taxonomic tiers. Furthermore, robust statistical testing was conducted to identify significant differential abundances between experimental groups at both the phylum and genus levels. To evaluate the structural disparities of the microbial communities among the groups, beta diversity was visualized via Principal Coordinate Analysis based on weighted UniFrac distance matrices. Concurrently, alpha diversity indices were calculated utilizing QIIME to accurately quantify the species richness and evenness within each individual sample (Kim et al., 2025).

**Table 1:** Sequence information statistics.

Sample name	Raw data	Clean data	Effective data	Q20	Q30	GC%	Ratio
HF.1	95,904	91,628	69,106	97.38	92.38	52.61	69.43
HF.2	91,196	87,404	65,655	97.47	92.5	52.6	69.49
HF.3	83,356	79,745	62,110	97.44	92.5	52.56	71.59
HF.4	76,243	72,297	55,492	97.15	91.81	52.68	68.99
HF.5	86,225	82,737	61,013	97.43	92.5	52.41	68.5
HM.1	80,591	74,386	69,470	97.19	91.82	53.42	60.95
HM.2	64,731	61,288	53,665	98.38	94.61	52.81	58.53
HM.3	77,829	75,171	68,070	98.04	93.93	56.02	75.07
HM.4	70,065	67,938	56,792	98.29	94.52	54.05	69.77
HM.5	82,660	76,164	66,252	97.9	93.41	53.23	62.12
NF.1	90,650	86,444	60,664	97.3	92.21	52.55	64.33
NF.2	83,651	78,372	55,769	96.96	91.42	52.26	62.62
NF.3	94,714	90,324	65,421	97.32	92.22	52.68	66.41
NF.4	95,472	91,285	67,102	97.32	92.2	52.51	67.46
NF.5	88,536	84,190	62,193	97.28	92.1	52.49	67.05
NF.6	90,308	85,789	59,850	97.14	91.85	52.84	63.12
NM.1	80,360	75,770	61,351	97.71	93.02	53.45	63.26
NM.2	53,832	50,780	43,882	98.1	93.98	52.68	60.41
NM.3	71,868	69,459	65,162	98.5	94.92	52.22	74.42
NM.4	76,889	74,499	61,577	97.41	92.32	53.18	59.77
NM.5	77,116	73,074	66,687	98.18	94.03	52.22	64.29

Raw Data, total number of raw sequencing reads obtained from the sequencing platform; Clean Data, number of reads retained after quality filtering and removal of low-quality sequences and adapters; Effective Data, number of high-quality valid reads used for downstream analysis; Q20, percentage of bases with a Phred quality score ≥ 20; Q30, percentage of bases with a Phred quality score ≥ 30; GC%, guanine–cytosine content of sequences; Ratio, percentage of effective reads relative to raw reads.

### 2.9 Microbial diversity, functional prediction, and integrated statistical analyses

To ensure unbiased comparisons across samples with variable library sizes, the OTU abundance table was rarefied to an even sequencing depth based on the sample with the lowest number of high-quality sequences prior to diversity analyses. Alpha diversity indices were calculated utilizing QIIME to accurately quantify species richness and evenness within each sample. Beta diversity was visualized via Principal Coordinate Analysis (PCoA) based on weighted UniFrac distance matrices to evaluate structural disparities of the microbial communities among groups. For downstream correlation networks and differential abundance testing, the OTU data were transformed into compositional relative abundances. Correlation analyses were performed to assess associations between lameness severity (locomotion score), production performance, metabolic parameters, inflammatory cytokines, and intestinal barrier-related indicators. Spearman correlation coefficients were calculated, and correlation heatmaps were generated for visualization. To characterize multivariate interactions, correlation network analysis was conducted using significant pairwise correlations (*p* < 0.05). Networks integrated variables across production, metabolic, inflammatory, intestinal barrier, and stress-related domains, and were visualized using Cytoscape software (version 3.10.2). Associations between microbial taxa and host traits were assessed at the genus level using Spearman correlation analysis. Significant correlations were visualized using heatmaps. Random forest models were constructed to identify microbial features discriminating between groups. Model performance was evaluated based on classification accuracy and variable importance (mean decrease in accuracy). Receiver operating characteristic (ROC) curves were generated using leave-one-out cross-validation (LOOCV), and the area under the curve (AUC) was calculated. Microbial co-abundance networks were constructed based on Spearman correlation analysis using genus-level relative abundance data. Pairwise correlations between taxa were calculated, and associations with |r| > 0.6 were retained to generate co-abundance networks. Modules of co-occurring taxa were identified using hierarchical clustering, and hub taxa within each module were defined based on network connectivity. Module–trait relationships were evaluated by correlating module abundance patterns with host production, metabolic, inflammatory, a.

nd intestinal barrier-related traits. Differential taxa were identified using linear discriminant analysis effect size (LEfSe), with significance defined as *p* < 0.05 and an LDA score > 2.0. Key taxa were further validated using Student’s t-test. Functional profiles were predicted using PICRUSt and annotated against the KEGG database at Level 2, Level 3, and KO levels. Differential pathways and orthologs were identified based on *p* < 0.05 and interpreted in the context of metabolic functions, including central carbon metabolism, transcription, transport, and carbohydrate utilization (Yang et al., 2025).

### 2.10 Statistical analyses

Statistical analyses were performed using GraphPad Prism 8 (GraphPad Software Inc., San Diego, CA, USA) and R software (version 4.2.3; R Foundation for Statistical Computing, Vienna, Austria). Data are presented as mean ± standard deviation (SD). Normality of data distribution was assessed using the Shapiro–Wilk test. Group comparisons of clinical and phenotypic parameters (e.g., serum biomarkers, production traits) were conducted using Student’s t-test for normally distributed data or the Mann–Whitney U test for non-normally distributed data, with statistical significance defined using raw *p*-values (*p* < 0.05). For correlation analyses, Spearman correlation coefficients were calculated depending on data distribution. To appropriately handle multiple comparisons inherent in high-dimensional microbiome data, the Benjamini–Hochberg false discovery rate (FDR) method was systematically applied. Specifically, FDR correction was performed for the pairwise Spearman correlation matrices and the PICRUSt-predicted functional pathway comparisons. These corrected values are explicitly reported as “FDR-adjusted *p*-values” throughout the results. For the LEfSe analysis, the standard pipeline was employed utilizing an alpha value of raw *p* < 0.05 for the factorial Kruskal-Wallis test, coupled with a stringent logarithmic LDA score threshold of > 2.0. To ensure maximum robustness, the key differential taxa initially identified via LEfSe were subsequently cross-validated using univariate tests subjected to the Benjamini-Hochberg FDR correction.

## 3 Results

### 3.1 Associations between lameness, production performance, and milk traits

**Figure 1:**
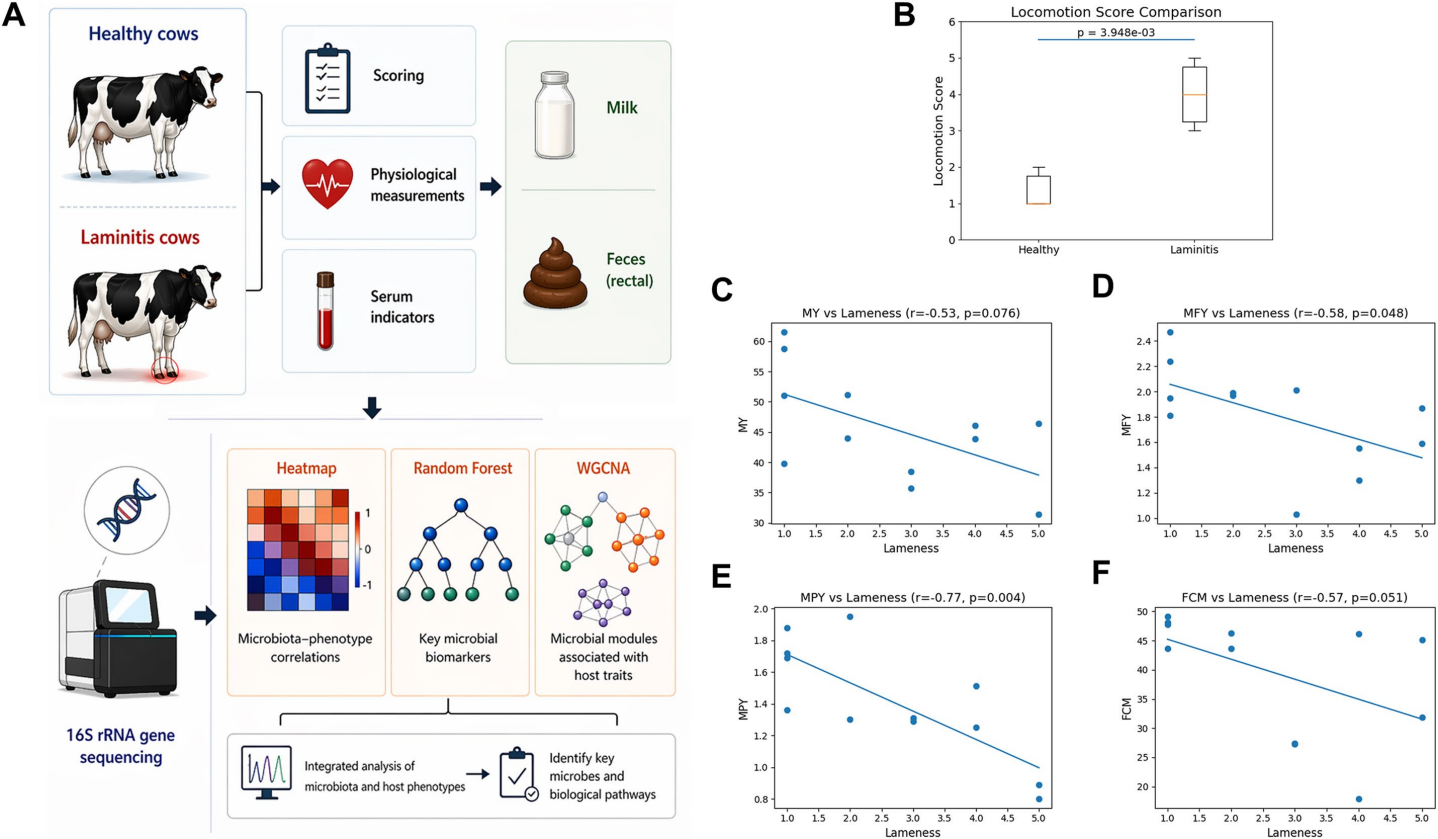
Associations between lameness, production performance, and milk traits. **(A)** Experimental design and analytical workflow. **(B)** Comparison of locomotion scores between healthy and laminitis cows. **(C)** Correlation between lameness score and milk yield (MY). **(D)** Correlation between lameness score and milk fat yield (MFY). **(E)** Correlation between lameness score and milk protein yield (MPY). **(F)** Correlation between lameness score and 3.5% fat-corrected milk (FCM).

Cows were classified into healthy and laminitis groups based on locomotion scoring, followed by sample collection, physiological measurements, serum analysis, and microbiota profiling using 16S rRNA gene sequencing (Figure 1A). Laminitis cows exhibited significantly higher locomotion scores than healthy controls (*p* = 3.95 × 10^−3^), confirming clear differences in clinical status (Figure 1B). No significant differences were observed between groups in milk composition traits, including MF, MP, ML, MSNF, MTS, MFP, MD, EC, and ash content (*p* > 0.05). In contrast, production performance was significantly impaired in laminitis cows. Milk yield (MY) (*p* = 0.029), milk fat yield (MFY) (*p* = 0.016), milk protein yield (MPY) (*p* = 0.012), energy-corrected milk (ECM) (*p* = 0.030), and 3.5% fat-corrected milk (FCM) (*p* = 0.013) were all significantly lower in the laminitis group. DMI and FE showed no significant differences, and no differences were observed in 305-day milk yield, historical production, BCS, parity, or DIM (*p* > 0.05) (Table 2). Correlation analysis further showed that milk production traits were negatively associated with lameness severity. MPY exhibited a strong negative correlation (*r* = −0.77, *p* = 0.004), and MFY showed a moderate negative correlation (r = −0.58, *p* = 0.048). MY and FCM also tended to decrease with increasing lameness score (MY: *r* = −0.53, *p* = 0.076; FCM: *r* = −0.57, *p* = 0.051), although these associations were not statistically significant (Figures 1C–F).

**Table 2:** Production performance and milk traits of healthy and laminitis cows.

Performance	Healthy	Laminitis	*p*-value
	Mean ± SEM	Mean ± SEM	
MF (%)	3.71 ± 0.11	3.86 ± 0.09	0.331
MP (%)	3.25 ± 0.06	3.37 ± 0.08	0.248
ML (%)	4.62 ± 0.20	4.87 ± 0.13	0.314
MSNF (%)	8.35 ± 0.36	8.54 ± 0.26	0.668
MTS (%)	13.08 ± 0.29	12.21 ± 0.43	0.127
MFP (°C)	−0.54 ± 0.01	−0.55 ± 0.01	0.425
MD (g/cm^3^)	1.02 ± 0.02	1.01 ± 0.03	0.82
EC (mS/cm)	5.12 ± 0.06	5.17 ± 0.14	0.762
Ash (%)	0.77 ± 0.04	0.78 ± 0.04	0.862
MY (kg/day)	51.03 ± 3.39	40.32 ± 2.50	0.029
MFY (kg/day)	2.07 ± 0.10	1.56 ± 0.15	0.016
MPY (kg/day)	1.65 ± 0.11	1.17 ± 0.11	0.012
ECM (kg/day)	62.79 ± 6.61	41.46 ± 5.20	0.03
3.5% FCM (kg/day)	46.44 ± 0.96	32.61 ± 4.52	0.013
DMI (kg/day)	22.95 ± 0.93	24.01 ± 0.74	0.391
FE (kg/kg)	1.43 ± 0.03	1.59 ± 0.08	0.083
305-d MY (kg/day)	43.91 ± 2.37	45.14 ± 1.62	0.679
Historical average 305-d MY (kg/day)	41.01 ± 1.08	43.62 ± 1.20	0.136
BCS	3.41 ± 0.15	3.27 ± 0.10	0.468
Parity	5.63 ± 0.22	5.39 ± 0.38	0.589
DIM (days)	126.56 ± 4.20	128.01 ± 3.72	0.802

MF, milk fat; MP, milk protein; ML, milk lactose; MSNF, milk solids-not-fat; MTS, milk total solids; MFP, milk freezing point; MD, milk density; EC, electrical conductivity; MY, milk yield; MFY, milk fat yield; MPY, milk protein yield; ECM, energy-corrected milk; FCM, fat-corrected milk (3.5%); DMI, dry matter intake; FE, feed efficiency; 305-d MY, 305-day milk yield; BCS, body condition score; DIM, days in milk.

Values are presented as mean ± SEM. Statistical significance was defined as *p* < 0.05.

### 3.2 Alterations in serum inflammatory, metabolic, and tissue degradation markers in laminitic dairy cows

Serum biochemical, metabolic, inflammatory, oxidative stress-related, and intestinal barrier-related variables were compared between healthy and laminitic cows. Most general biochemical parameters, including TP, ALB, GLB, ALB/GLB, ALT, AST, ALP, *γ*-GGT, TBA, BUN, CRE, GLU, NEFA, BHB, GSX-PX, T-AOC, T-GSH, IgG, IgA, HPT, and SAA, did not differ significantly between groups (*p* > 0.05). In contrast, laminitic cows showed significant changes in several serum parameters. LDH activity (*p* = 0.018), cortisol (*p* = 0.012), histamine (*p* = 0.019), and MDA (*p* = 0.033) were significantly increased, whereas TC (*p* = 0.028), TG (*p* = 0.015), SOD (*p* = 0.013), and CAT (*p* = 0.019) were significantly reduced. Inflammatory cytokines, including IL-1β (*p* = 0.036), IL-6 (*p* = 0.048), IL-10 (*p* = 0.010), and TNF-*α* (*p* = 0.024), were significantly elevated in laminitic cows. In addition, intestinal barrier- and tissue damage-related markers, including LPS (*p* = 0.019), DAO (*p* = 0.030), D-lactate (*p* = 0.012), and MMP-9 (*p* = 0.022), were significantly increased. These results indicate that laminitis is associated with systemic inflammatory responses, oxidative imbalance, and altered intestinal barrier-related indicators (Table 3).

**Table 3:** Comparison of serum biochemical, oxidative stress, inflammatory, and barrier function parameters between healthy and laminitic cows.

Performance	Healthy	Laminitis	*p*-value
	Mean ± SEM	Mean ± SEM	
TP (g/L)	74.37 ± 1.84	75.32 ± 2.05	0.737
ALB (g/L)	41.94 ± 1.57	41.55 ± 0.77	0.828
GLB (g/L)	28.72 ± 0.66	30.03 ± 0.68	0.198
ALB/GLB	1.36 ± 0.25	1.28 ± 0.19	0.785
ALT (U/L)	41.63 ± 2.71	42.87 ± 1.86	0.716
AST (U/L)	81.95 ± 4.81	83.78 ± 4.71	0.792
ALP (U/L)	237.80 ± 28.36	250.35 ± 34.87	0.786
TBA (μmol/L)	5.17 ± 1.14	5.83 ± 1.09	0.685
γ-GGT (U/L)	27.36 ± 5.65	32.89 ± 5.53	0.5
BUN (mmol/L)	4.66 ± 0.72	5.63 ± 0.93	0.427
CRE (μmol/L)	78.17 ± 7.01	81.24 ± 5.35	0.735
LDH (U/L)	655.76 ± 19.42	754.41 ± 28.94	0.018
GLU (mmol/L)	4.61 ± 0.48	3.96 ± 0.32	0.286
TC (mmol/L)	4.31 ± 0.14	3.80 ± 0.14	0.028
TG (mmol/L)	0.56 ± 0.03	0.45 ± 0.02	0.015
HDL-C (mmol/L)	1.91 ± 0.04	1.86 ± 0.10	0.676
LDL-C (mmol/L)	2.01 ± 0.14	1.66 ± 0.18	0.152
NEFA (μmol/L)	381.32 ± 45.58	464.92 ± 25.90	0.142
BHB (μmol/L)	737.14 ± 31.02	744.21 ± 24.76	0.862
SOD (U/mL)	110.55 ± 2.53	100.46 ± 2.17	0.013
GSX-PX (U/mL)	59.42 ± 0.87	59.09 ± 1.90	0.877
T-AOC (mmol/L)	1.95 ± 0.07	1.93 ± 0.08	0.828
T-GSH (μmol/L)	3.57 ± 0.11	3.49 ± 0.07	0.537
CAT (U/mL)	6.21 ± 0.26	4.78 ± 0.45	0.019
MDA (μmol/L)	3.56 ± 0.06	3.76 ± 0.05	0.033
PC (μmol/L)	1.65 ± 0.11	1.69 ± 0.05	0.757
IL-1β (pg/mL)	8.65 ± 0.52	10.23 ± 0.39	0.036
IL-6 (pg/mL)	18.31 ± 0.57	20.65 ± 0.87	0.048
IL-10 (pg/mL)	9.33 ± 0.53	12.48 ± 0.85	0.01
HPT (μg/mL)	38.51 ± 4.81	49.25 ± 7.39	0.251
TNF-α (pg/mL)	13.92 ± 0.36	15.81 ± 0.61	0.024
SAA (μg/mL)	25.75 ± 1.47	27.52 ± 2.37	0.54
IgG (g/L)	23.07 ± 0.39	23.32 ± 0.27	0.603
IgA (g/L)	0.63 ± 0.06	0.66 ± 0.05	0.714
LPS (EU/mL)	0.37 ± 0.13	0.78 ± 0.06	0.019
DAO (U/L)	3.28 ± 0.93	6.46 ± 0.84	0.03
D-Lactate (mmol/L)	0.31 ± 0.05	0.62 ± 0.09	0.012
MMP-9 (ng/mL)	52.56 ± 11.43	91.53 ± 8.80	0.022
Histamine (nmol/L)	5.83 ± 0.77	9.47 ± 1.05	0.019
Cortisol (ng/mL)	14.61 ± 1.43	21.25 ± 1.62	0.012

TP, total protein; ALB, albumin; GLB, globulin; ALB/GLB, albumin-to-globulin ratio; ALT, alanine aminotransferase; AST, aspartate aminotransferase; ALP, alkaline phosphatase; TBA, total bile acids; γ-GGT, gamma-glutamyl transferase; BUN, blood urea nitrogen; CRE, creatinine; LDH, lactate dehydrogenase; GLU, glucose; TC, total cholesterol; TG, triglycerides; HDL-C, high-density lipoprotein cholesterol; LDL-C, low-density lipoprotein cholesterol; NEFA, non-esterified fatty acids; BHB, β-hydroxybutyrate; SOD, superoxide dismutase; GSX-PX (GSH-Px), glutathione peroxidase; T-AOC, total antioxidant capacity; T-GSH, total glutathione; CAT, catalase; MDA, malondialdehyde; PC, protein carbonyl; IL-1β, interleukin-1 beta; IL-6, interleukin-6; IL-10, interleukin-10; HPT, haptoglobin; TNF-α, tumor necrosis factor-alpha; SAA, serum amyloid A; IgG, immunoglobulin G; IgA, immunoglobulin A; LPS, lipopolysaccharide; DAO, diamine oxidase; MMP-9, matrix metalloproteinase-9.

Values are expressed as mean ± SEM. Statistical significance was defined as *p* < 0.05.

### 3.3 Integrated correlations among lameness severity, production performance, inflammation, and intestinal barrier function

**Figure 2:**
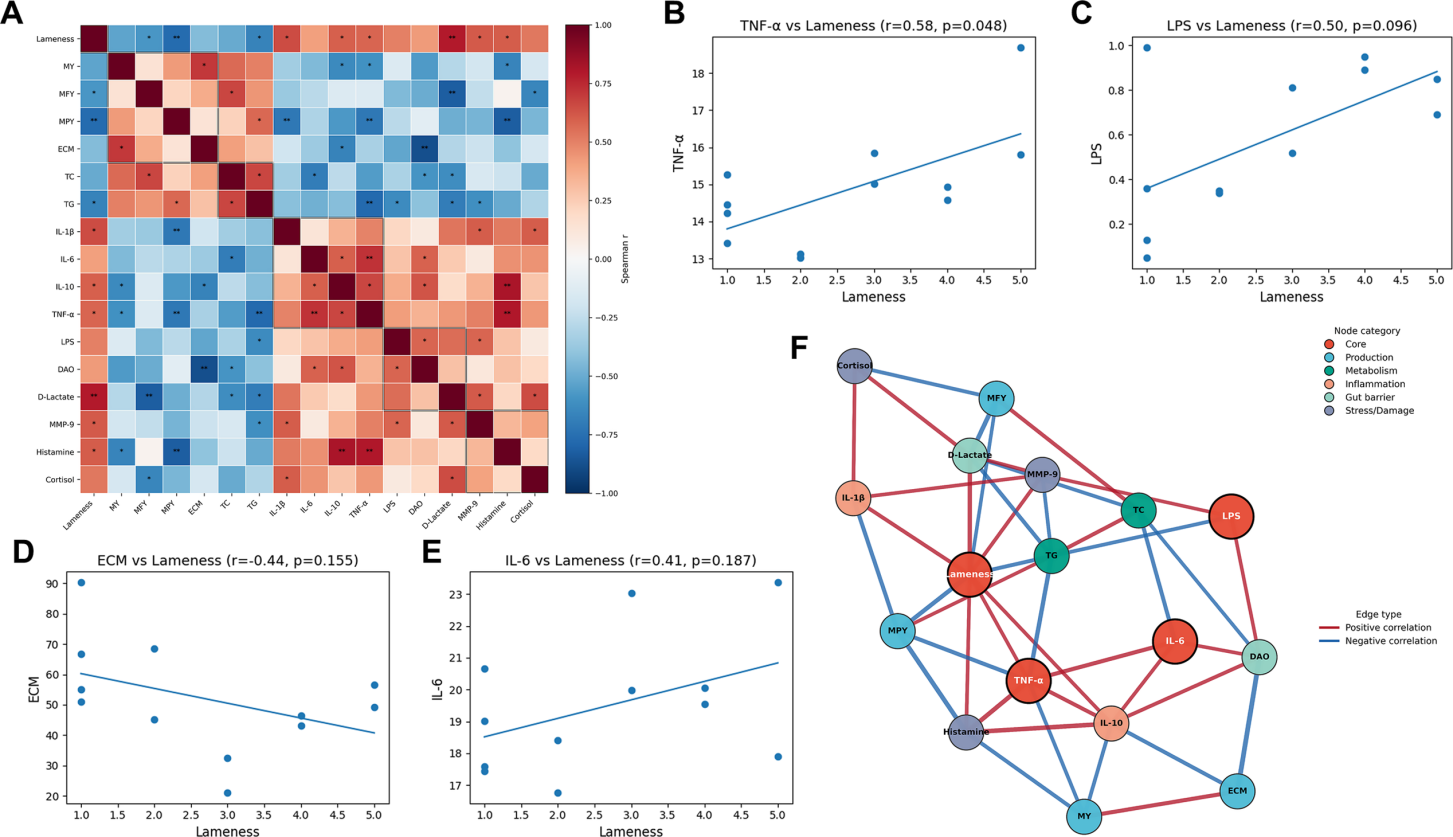
Integrated correlations among lameness severity, production performance, inflammation, and intestinal barrier function. **(A)** Correlation heatmap of significantly changed variables. Spearman correlation analysis was performed among locomotion score, milk production traits (MY, MFY, MPY, ECM), metabolic indicators (TC, TG), inflammatory markers (IL-1*β*, IL-6, IL-10, TNF-*α*), and intestinal barrier-related variables (LPS, DAO, D-lactate, MMP-9, histamine, cortisol). Color scale represents correlation coefficients (r), with red indicating positive correlations and blue indicating negative correlations. Asterisks indicate statistically significant correlations (*p < 0.05, **p < 0.01). **(B)** Correlation between lameness score and TNF-α. **(C)** Correlation between lameness score and LPS. **(D)** Correlation between lameness score and energy-corrected milk (ECM). **(E)** Correlation between lameness score and interleukin-6 (IL-6). **(F)** Correlation network of clinical, production, metabolic, inflammatory, intestinal barrier, and stress-related variables. Nodes represent variables and are colored according to their categories (core, production, metabolism, inflammation, gut barrier, and stress/damage). Edges represent significant correlations between variables, with red lines indicating positive correlations and blue lines indicating negative correlations.

Correlation analysis was performed using significantly altered clinical, production, metabolic, inflammatory, and intestinal barrier-related variables. Lameness score was negatively correlated with milk production traits, including MY, MFY, MPY, and ECM, but positively correlated with inflammatory cytokines (IL-1β, IL-6, IL-10, and TNF-α) and intestinal barrier-related markers (LPS, DAO, D-lactate, and MMP-9). Milk production traits were generally negatively associated with inflammatory and barrier-related markers, whereas inflammatory cytokines showed positive correlations with intestinal barrier indicators (Figure 2A). Consistent with these patterns, lameness score was significantly positively correlated with TNF-*α* (*r* = 0.58, *p* = 0.048) and showed a positive trend with LPS (*r* = 0.50, *p* = 0.096). Lameness score was also negatively correlated with ECM (*r* = −0.44, *p* = 0.155) and positively correlated with IL-6 (*r* = 0.41, *p* = 0.187), although these associations were not statistically significant (Figures 2B–E). Correlation network analysis further showed that lameness score was connected with production traits (MY, MFY, and MPY), metabolic indicators (TG and TC), inflammatory markers (IL-1β, IL-6, IL-10, and TNF-α), intestinal barrier-related variables (LPS, DAO, D-lactate, and MMP-9), and stress-related factors (cortisol and histamine). Production traits were mainly negatively associated with inflammatory and barrier-related variables, while inflammatory markers showed extensive positive links with intestinal barrier indicators. Overall, these results indicate coordinated relationships among lameness severity, reduced production performance, metabolic alterations, inflammatory response, and intestinal barrier-related changes (Figure 2F).

### 3.4 Sequencing quality and microbial community structure across groups

**Figure 3:**
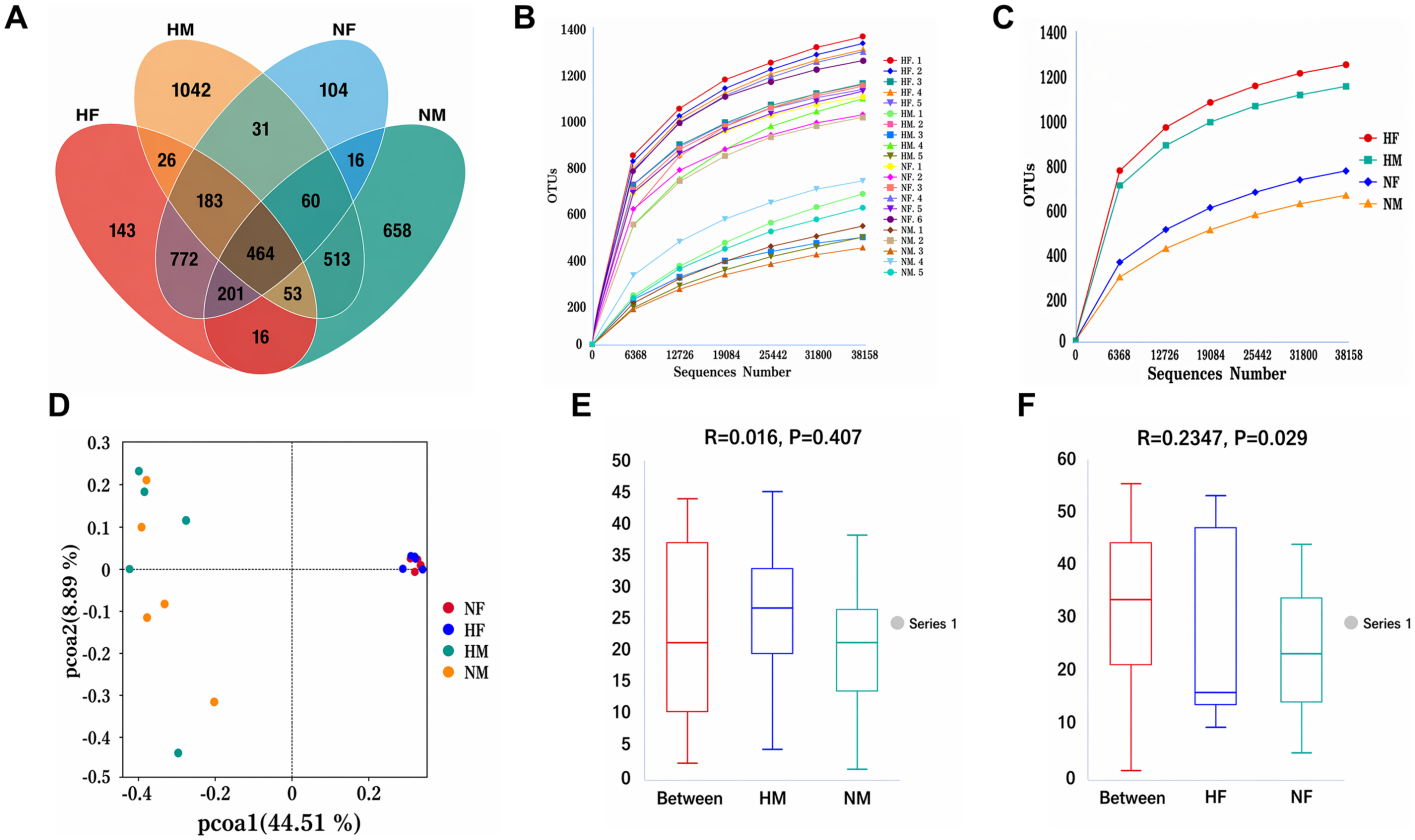
Sequencing quality and microbial community structure across groups. **(A)** Venn diagram showing shared and unique OTUs among HF, NF, HM, and NM groups. **(B)** Rarefaction curves of individual samples based on sequencing depth. **(C)** Rarefaction curves at the group level. **(D)** Principal coordinate analysis (PCoA) of samples based on distance matrix. **(E)** Comparison of group differences between HM and NM. **(F)** Comparison of group differences between HF and NF.

High-throughput 16S rRNA gene sequencing generated 1,712,196 raw reads, of which 1,297,283 high-quality valid sequences were retained after quality control and sequence optimization. Sequencing quality was high across samples, with Q20 and Q30 values of approximately 97 and 92%, respectively, and GC contents ranging from 58.53 to 75.07% (Table 1). Shared and unique OTUs were then assessed to compare microbial composition among groups. The Venn diagram showed both common and group-specific OTUs across HF, NF, HM, and NM groups, indicating partial overlap and group-specific microbial features (Figure 3A). Rarefaction curves gradually approached a plateau with increasing sequencing depth, suggesting sufficient sequencing coverage for microbial diversity analysis. At the group level, HF and HM generally showed higher OTU richness than NF and NM (Figures 3B,C). PCoA was further performed to evaluate overall microbial community structure. Samples showed distinct clustering patterns among NF, HF, HM, and NM groups (Figure 3D). ANOSIM was performed to assess differences in microbial community structure between groups. For milk microbiota, no significant difference was observed between HM and NM groups (*R* = 0.016, *p* = 0.407; Figure 3E), indicating similar overall community composition. In contrast, fecal microbiota differed significantly between HF and NF groups (*R* = 0.2347, *p* = 0.029; Figure 3F). It is worth noting that while the 97% OTU clustering approach utilized here effectively captures broad taxonomic shifts at the community level, it may lack the fine-scale resolution of modern Amplicon Sequence Variant (ASV) methods for detecting strain-level microdiversity, which is acknowledged as a methodological limitation of the present study. These results indicate that laminitis was associated with a significant shift in fecal microbial community structure, whereas no comparable overall change was detected in milk microbiota (Figures 3E,F).

### 3.5 Alpha diversity differences in fecal and milk microbiota

**Figure 4:**
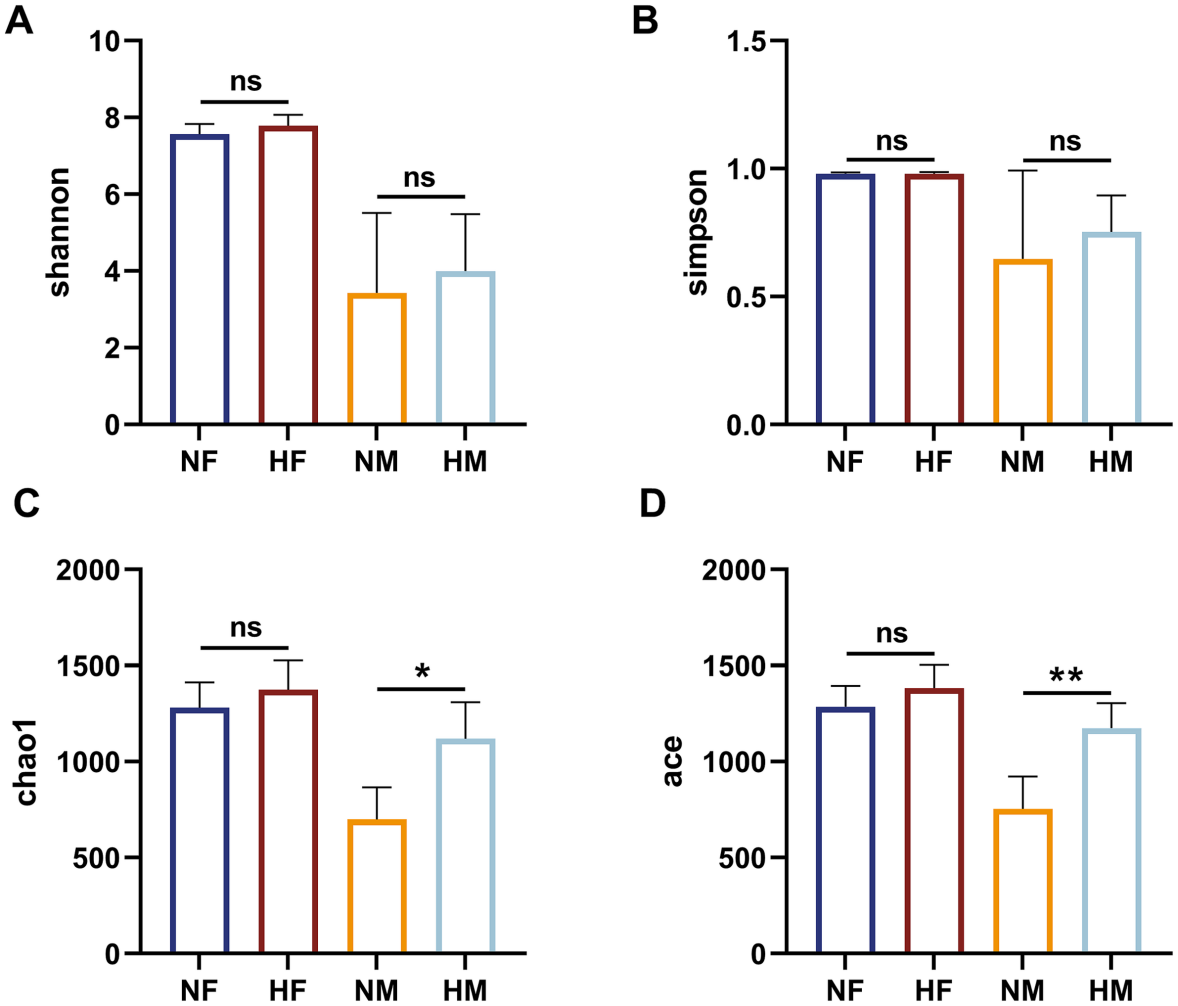
Alpha diversity differences in fecal and milk microbiota. **(A)** Comparison of Shannon diversity index among HF, NF, HM, and NM groups. **(B)** Comparison of Simpson diversity index among HF, NF, HM, and NM groups. **(C)** Comparison of Chao1 richness index among HF, NF, HM, and NM groups. **(D)** Comparison of ACE richness index among HF, NF, HM, and NM groups. *P < 0.05, **P < 0.01.

Alpha diversity was assessed using the Shannon, Simpson, Chao1, and ACE indices (Figures 4A–D). Shannon and Simpson indices showed slight increases in both HF and HM groups compared with their corresponding controls (NF and NM), but these differences were not statistically significant (Figures 4A,B). Richness estimates based on Chao1 and ACE showed a similar non-significant increase in HF compared with NF. In contrast, both Chao1 and ACE indices were significantly higher in HM than in NM (Chao1: *p* < 0.05; ACE: *p* < 0.01; Figures 4C,D). These results indicate that overall microbial diversity was largely unchanged, whereas microbial richness was increased in milk samples from laminitic cows.

### 3.6 Taxonomic composition of milk and fecal microbiota

**Figure 5:**
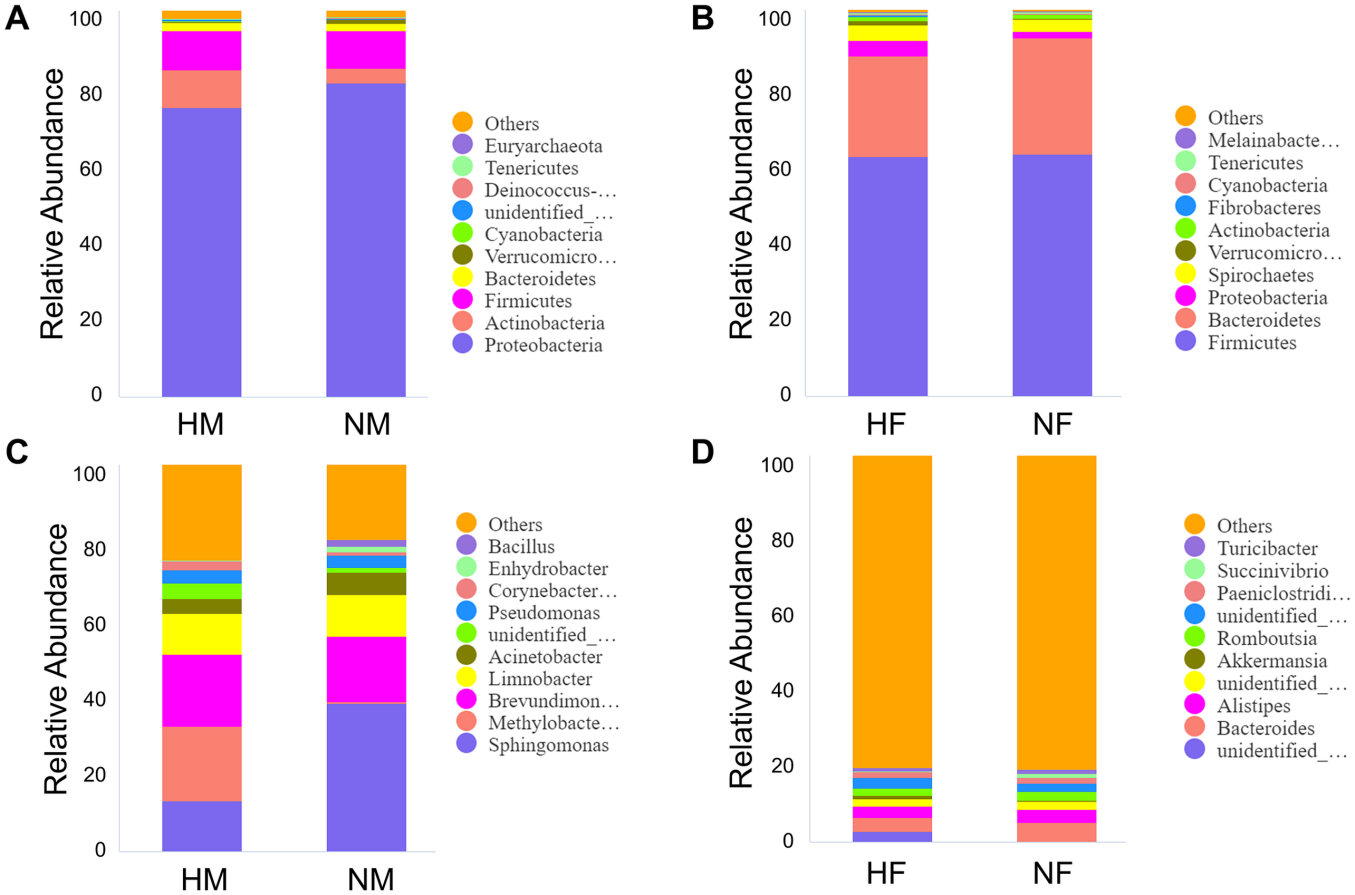
Taxonomic composition of milk and fecal microbiota. **(A)** Relative abundance analysis of bacteria in milk from healthy and cows with laminitis at the phylum level. **(B)** Relative abundance analysis of bacteria in fecal samples from healthy and cows with laminitis at the phylum level. **(C)** Relative abundance analysis of bacteria in milk from healthy and cows with laminitis at the genus level. **(D)** Relative abundance analysis of bacteria in fecal samples from healthy and cows with laminitis at the genus level.

Microbial taxonomic composition was evaluated at the phylum and genus levels in milk and fecal samples. At the phylum level, milk microbiota was mainly dominated by *Proteobacteria, Actinobacteria, Firmicutes, and Bacteroidetes* (Figure 5A). Compared with the HM group, the NM group showed a higher relative abundance of *Proteobacteria, whereas Actinobacteria* was more abundant in the HM group. Minor phyla, including *Verrucomicrobia and Deinococcus-Thermus*, were also relatively more abundant in the NM group. In fecal samples, *Firmicutes* and *Bacteroidetes* were the predominant phyla, followed by Proteobacteria (Figure 5B). Compared with milk samples, fecal microbial composition showed greater similarity between HF and NF groups, with no major shifts in dominant phyla.

Genus-level profiling further showed taxonomic differences between milk and fecal microbiota. In milk samples, *Methylobacterium* was enriched in the HM group, whereas *Sphingomonas* was more abundant in the NM group (Figure 5C). Other genera, including *Brevundimonas, Limnobacter, and Acinetobacter*, showed relatively stable abundance between groups. In fecal samples, the dominant genera in both HF and NF groups included *Bacteroides, Alistipes, Romboutsia, and unclassified Clostridiales* (Figure 5D). Overall, the major fecal genera were relatively consistent between HF and NF groups, whereas milk microbiota showed more evident taxonomic variation between HM and NM groups.

### 3.7 Associations between microbial genera, host traits, and lameness-related status

**Figure 6:**
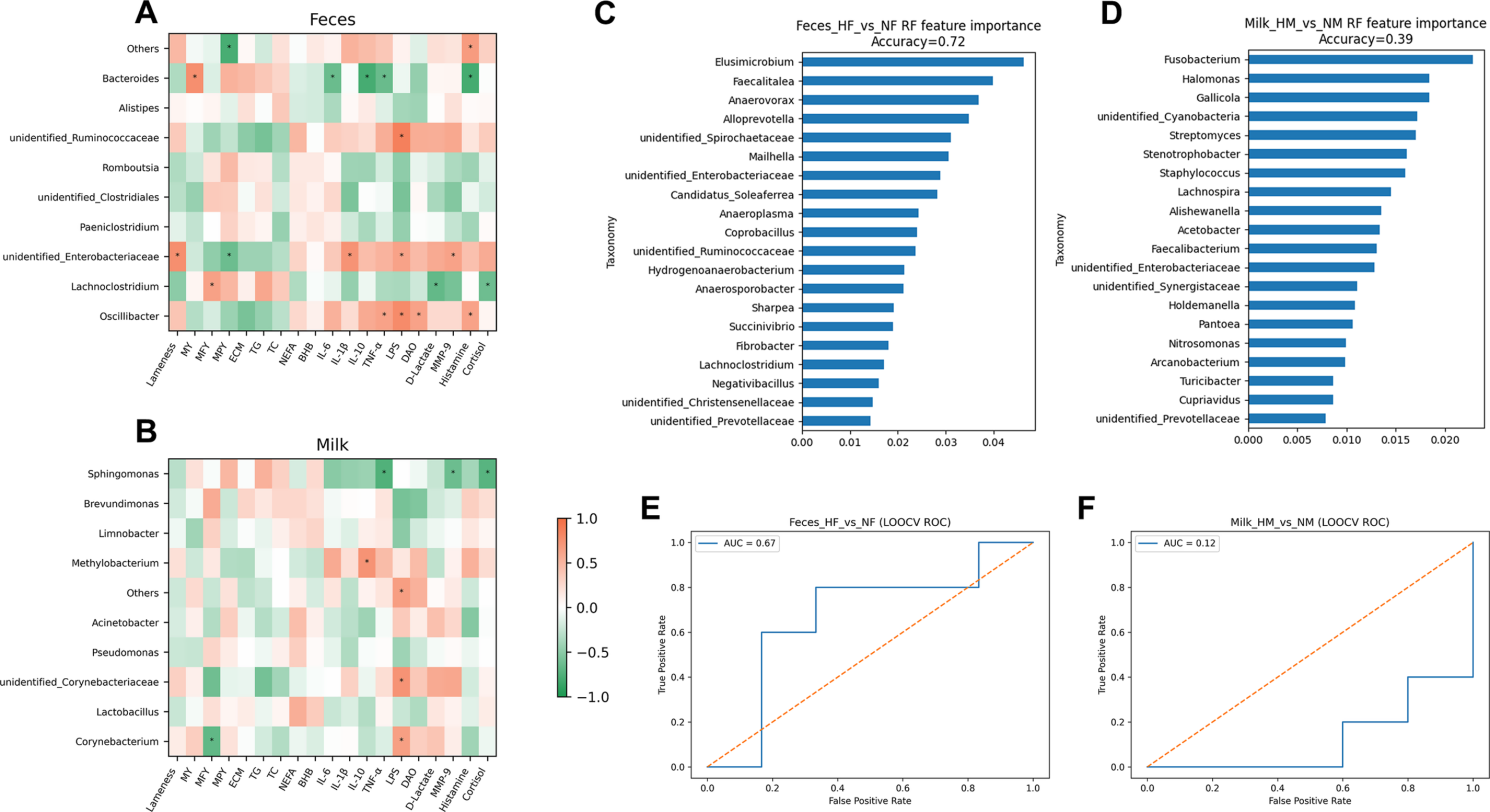
Associations between microbial genera, host traits, and lameness-related status. **(A)** Heatmap showing Spearman correlations between bacterial genera and host-related parameters, including lameness score, production traits, metabolic indicators, and inflammatory markers, in fecal samples. **(B)** Heatmap showing Spearman correlations between bacterial genera in milk samples and host-related parameters, including lameness score, production traits, metabolic indicators, and inflammatory markers. **(C)** Random forest analysis showing the importance of bacterial genera for distinguishing HF and NF groups based on fecal microbiota. **(D)** Random forest analysis showing the importance of bacterial genera for distinguishing HM and NM groups based on milk microbiota. **(E)** ROC curve for classification of HF and NF groups based on microbial features using LOOCV. **(F)** ROC curve for classification of HM and NM groups based on microbial features using LOOCV.

Genus-level correlation analysis was performed to examine associations between microbial taxa and host traits. In fecal samples, multiple genera were associated with lameness score, production traits, metabolic indicators, and inflammatory markers. Several genera showed correlations with lameness-related inflammatory and barrier-associated variables, including IL-6, TNF-α, and LPS, while others were associated with production traits such as MY, MFY, and MPY (Figure 6A). Similar analyses in milk samples also identified associations between bacterial genera and host variables, although the correlation patterns differed from those observed in fecal microbiota (Figure 6B). As an exploratory approach, random forest analysis was applied to evaluate potential microbial taxa contributing to group separation. In fecal samples, the model distinguished HF from NF with an accuracy of 0.72. The most important genera included *Elusimicrobium*, *Faecalitalea*, *Anaerovorax*, *Alloprevotella*, and *unidentified_Spirochaetaceae* (Figure 6C). In milk samples, the model showed lower classification accuracy for HM versus NM (accuracy = 0.39), with *Fusobacterium*, *Halomonas*, *Gallicola*, and *unidentified_Cyanobacteria* ranking among the most important features (Figure 6D). Model performance was further evaluated using LOOCV-based ROC analysis. It is crucial to note that due to the extremely small sample size, these machine learning analyses are strictly exploratory. Under these constraints, the fecal microbiota model showed moderate, albeit preliminary, discrimination between the HF and NF groups (AUC = 0.67) (Figure 6E). Conversely, the milk microbiota model failed to establish a reliable predictive signature between the HM and NM groups. The observed AUC of 0.12 and classification accuracy of 0.39 fall below random chance, indicating severe model instability and confirming that no robust predictive microbial pattern could be extracted from the milk samples (Figure 6F). Consequently, the identified variable importance features should be interpreted merely as descriptive taxonomic differences within this specific cohort rather than true diagnostic biomarkers. Overall, these results indicate that fecal microbiota showed stronger exploratory associations with lameness-related status than milk microbiota.

### 3.8 Microbial co-abundance networks and module–trait associations

**Figure 7:**
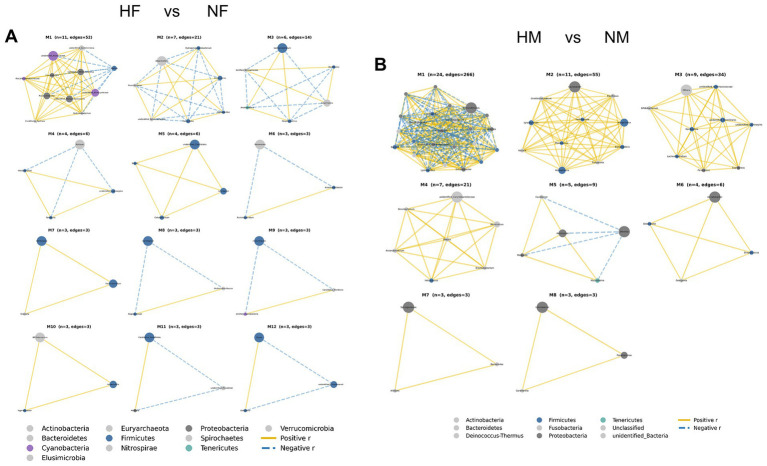
Microbial co-abundance network modules across fecal and milk microbiota. **(A)** Microbial co-abundance network modules of fecal microbiota in HF versus NF groups based on Spearman correlations (|r| > 0.6), showing modular structures (M1–M12) and interaction patterns among bacterial genera. **(B)** Microbial co-abundance network modules of milk microbiota in HM versus NM groups based on Spearman correlations (|r| > 0.6), showing modular organization (M1–M8) and interaction patterns among bacterial genera.

Co-abundance network analysis was performed to characterize microbial interaction patterns in fecal and milk microbiota based on Spearman correlations (|*r*| > 0.6). In the HF versus NF fecal comparison, 12 microbial modules were identified. Among them, Module 1 (M1) was the largest and most complex subnetwork, consisting of 11 genera and 52 edges. Several genera showed high connectivity within M1, including *Candidatus_Methylomirabilis, unidentified_Microcystaceae, Limnohabitans*, and *Polynucleobacter*, indicating their potential roles as hub taxa (Figure 7A). In the HM versus NM milk comparison, eight modules were identified. M1 was also the largest and most densely connected subnetwork, containing 24 genera and 266 edges. Hub genera in this module included *Lactobacillus, Bacteroides, Faecalibacterium, Prevotella, and Ruminococcus* (Figure 7B). Compared with the fecal network, the milk M1 module showed a larger size and higher connectivity, indicating differences in microbial network organization between sample types.

**Figure 8:**
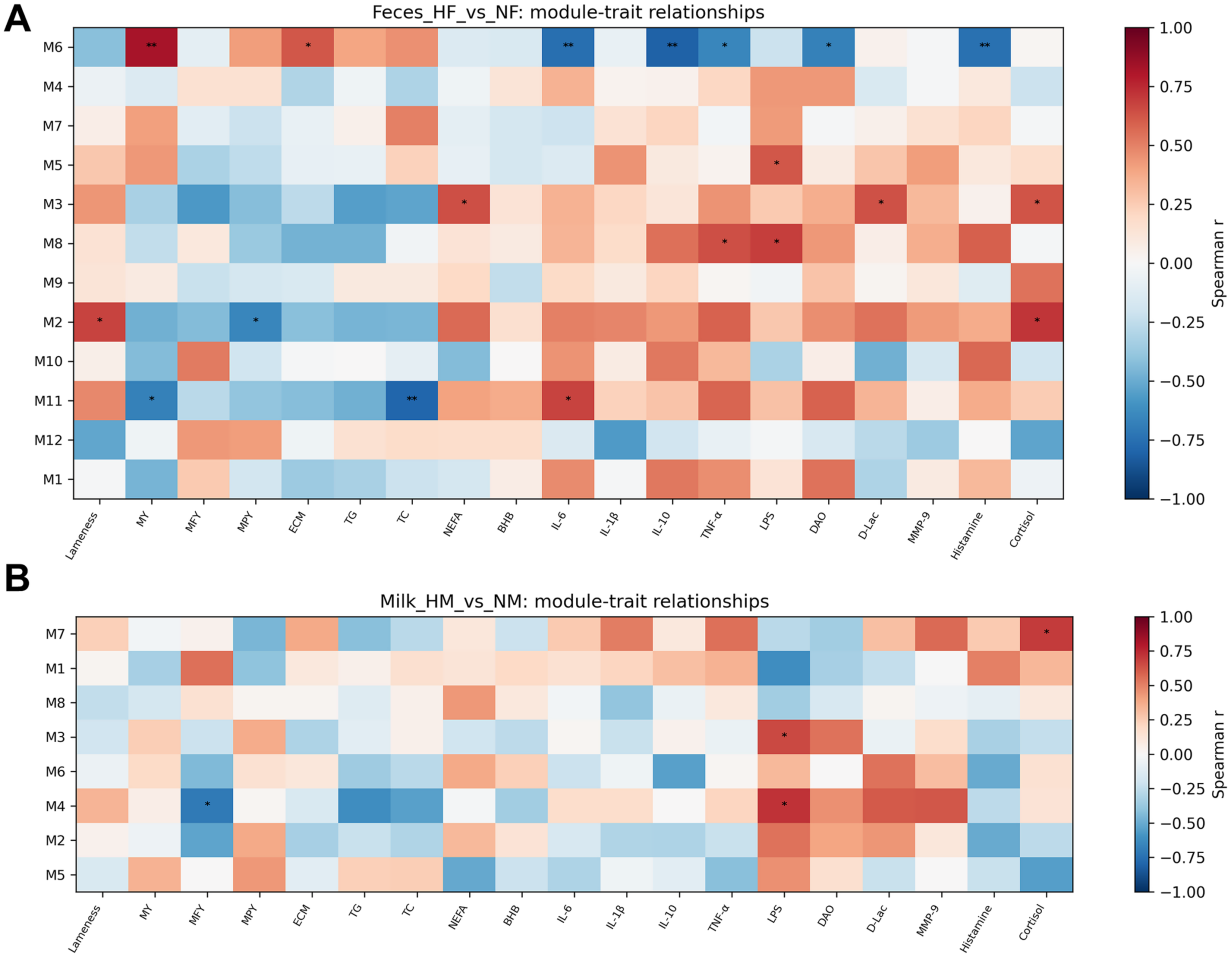
Module–trait relationships between microbial co-abundance modules and host variables. **(A)** Module–trait relationships in fecal microbiota of HF versus NF groups based on Spearman correlations, showing associations between microbial modules and host phenotypic, metabolic, and inflammatory variables. **(B)** Module–trait relationships in milk microbiota of HM and NM groups based on Spearman correlations. *P < 0.05, **P < 0.01.

Module–trait correlation analysis further showed that several fecal microbial modules were associated with host clinical and physiological traits. Some modules were positively correlated with inflammatory markers, including IL-6, TNF-*α*, and IL-10, as well as intestinal barrier-related indicators such as LPS and D-lactate. In contrast, these modules tended to be negatively correlated with production traits, including MY, MFY, MPY, and ECM. Associations with metabolic indicators, including TC, TG, and NEFA, were also observed (Figure 8A). In milk samples, module–trait associations were also detected, although the correlations were generally less consistent across modules. Several modules were related to inflammatory, gut barrier-related, metabolic, and production traits, indicating moderate associations between milk microbial modules and host physiological parameters (Figure 8B).

### 3.9 Differential microbial biomarkers and predicted functional profiles associated with laminitis

**Figure 9:**
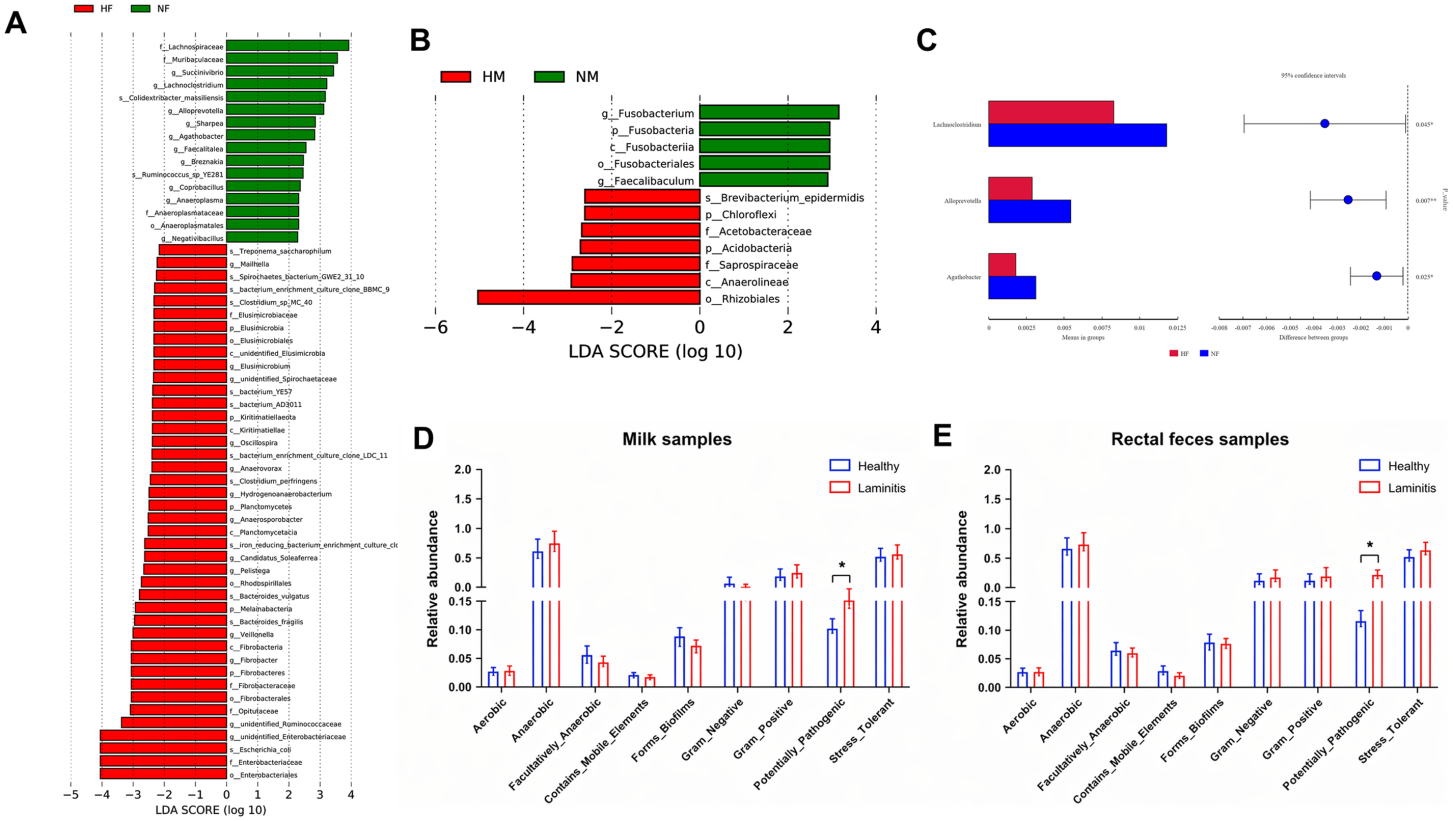
Differential microbial biomarkers and predicted functional profiles associated with laminitis. **(A)** LDA value distribution histogram of fecal samples from healthy cows and cows with laminitis. **(B)** LDA value distribution histogram of milk samples from healthy cows and cows with laminitis. **(C)** Genus-level comparison of differential taxa between healthy and laminitic cows in fecal samples. **(D,E)** Predicted functional profiles of microbial communities in milk and fecal samples, comparing healthy and laminitis groups.

LEfSe analysis was performed to identify microbial biomarkers that differed between healthy and laminitis groups. In fecal samples, distinct taxonomic signatures were observed between HF and NF groups (Figure 9A; Supplementary Figure S1A). The HF group was enriched in several taxa, including the genera *Mailhella* and *Oscillospira*, the species *Treponema saccharophilum* and *Colidextribacter massiliensis*, as well as the order *Enterobacterales* and family *Enterobacteriaceae*. In contrast, the NF group was characterized by higher abundance of *Alloprevotella, Sharpea, Agathobacter*, members of *Lachnospiraceae and Muribaculaceae, Ruminococcus* sp. *YE281*, and *Succinivibrio*. The cladogram further showed that these differential taxa were di.stributed across multiple taxonomic levels. LEfSe analysis of milk microbiota identified a different set of biomarkers between HM and NM groups (Figure 9B; Supplementary Figure S1B). In the NM group, *Faecalibaculum* and *Fusobacterium* were identified as enriched taxa. In the HM group, *Brevibacterium epidermidis* was identified as a species-level biomarker. At higher taxonomic levels, the HM group showed enrichment of *Chloroflexi, Acidobacteria, Anaerolineae, Rhizobiales*, *Acetobacteraceae*, and *Saprospiraceae*, whereas the NM group was enriched in *Fusobacteria* and *Fusobacteriales.* These results indicate that fecal and milk microbiota showed distinct group-associated taxonomic signatures. To further validate differential taxa in fecal microbiota, genus-level comparisons were performed using Student’s t-test (Figure 9C). Alloprevotella was significantly reduced in the HF group compared with the NF group (FDR-adjusted *p* < 0.01). Agathobacter and Lachnoclostridium also differed significantly between groups (both FDR-adjusted *p* < 0.05). All three genera showed lower relative abundance in the HF group, with *Lachnoclostridium* showing the largest absolute difference in mean abundance.

Functional prediction analysis was then used to compare microbial functional profiles between healthy and laminitis groups in milk and fecal samples. Most predicted functional categories were comparable between groups, although some functions, including potentially pathogenic traits, differed between healthy and laminitis groups in both sample types. Overall, the predicted functional profiles showed limited variation between groups (Figures 9D,E).

### 3.10 predicted functional alterations in fecal microbiota associated with laminitis

**Figure 10:**
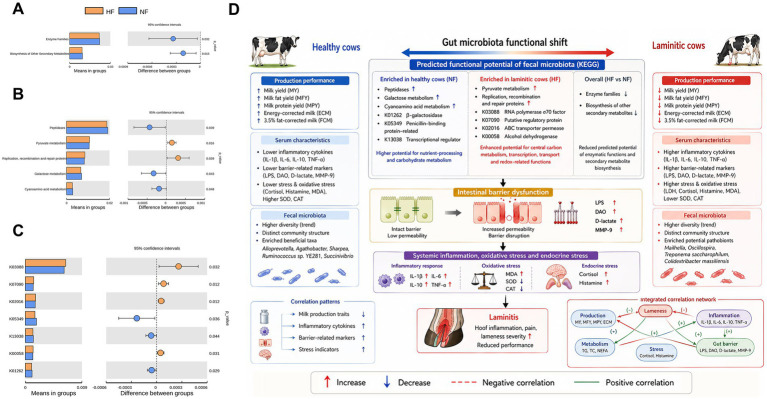
Predicted functional alterations in fecal microbiota associated with laminitis. **(A)** Differential predicted KEGG Level 2 pathways between HF and NF groups. **(B)** Differential predicted KEGG Level 3 pathways between HF and NF groups. **(C)** Differential KEGG Orthology profiles between HF and NF groups **(D)** Schematic diagram of predicted gut microbiome metabolic shifts associated with laminitis.

PICRUSt-based functional prediction was performed to evaluate functional differences in fecal microbiota between HF and NF groups. At KEGG Level 2, pathways related to enzyme families (FDR-adjusted *p* = 0.032) and biosynthesis of other secondary metabolites (FDR-adjusted *p* = 0.015) were significantly reduced in the HF group compared with the NF group, indicating a lower inferred relative abundance of genes associated with enzymatic and secondary metabolite biosynthetic pathways in fecal microbiota from laminitic cows (Figure 10A). At KEGG Level 3, the predictive pathway for pyruvate metabolism was significantly enriched in the HF group (FDR-adjusted *p* = 0.016), together with pathways related to replication, recombination, and repair proteins (FDR-adjusted *p* = 0.039). In contrast, peptidases (FDR-adjusted *p* = 0.039), galactose metabolism (FDR-adjusted *p* = 0.043), and cyanoamino acid metabolism (FDR-adjusted *p* = 0.048) were more abundant in the NF group (Figure 10B). These results suggest that the fecal microbiota from laminitic cows exhibited an enriched predicted genetic potential for central carbon metabolism and genomic maintenance, while healthy cows possessed a higher predicted genomic capacity for nutrient-processing and carbohydrate metabolism. At the KEGG Orthology level, several orthologs differed significantly between groups. K03088 (RNA polymerase sigma-70 factor), K07090 (putative regulatory protein), K02016 (ABC transporter permease protein), and K00058 (alcohol dehydrogenase) were enriched in the HF group (FDR-adjusted *p* < 0.05), which are computationally mapped to functional pathways related to transcription, regulation, transport, and redox metabolism. In contrast, K05349 (penicillin-binding protein-related), K13038 (transcriptional regulator), and K01262 (beta-galactosidase) were enriched in the NF group (FDR-adjusted *p* < 0.05), mainly associated with cell wall-related processes, transcriptional regulation, and carbohydrate degradation (Figure 10C). Overall, the model summarizes a potential link between the altered predicted genomic capacities of the gut microbiota and laminitis-associated physiological changes (Figure 10D). It must be emphasized that these PICRUSt-derived profiles reflect inferred genomic potential rather than actual *in vivo* expressed functions, necessitating future metatranscriptomic or metabolomic validation.

## 4 Discussion

Laminitis is closely associated with coordinated disruptions in systemic health, specifically involving intestinal and mammary physiological functions. While milk composition remained stable, the marked reduction in production performance indicates compromised mammary function. Concurrently, observed intestinal barrier dysfunction and microbial shifts point to disturbed gut homeostasis. These localized changes coincided with systemic inflammation and oxidative imbalance. Ultimately, these findings support a model wherein laminitis is linked to a gut–systemic inflammatory disruption, which correlates with metabolic imbalances and secondary impairments in mammary physiological function and overall productivity.

From the perspective of disease progression, our findings suggest that the clinical manifestation of laminitis is closely linked to a state of systemic stress that occurs concurrently with disruptions in intestinal and mammary function (Tuniyazi et al., 2024). The observed reduction in milk yield, despite stable composition, reflects impaired mammary performance likely associated with pain, altered feeding behavior, and stress (Espiritu et al., 2025; Palhano et al., 2024). Concurrently, elevated circulating LPS, DAO, and D-lactate, alongside inflammatory markers, indicate compromised intestinal barrier function. While our cross-sectional design precludes causal inferences, pain and reduced feed intake efficiency during laminitis may negatively influence mucosal integrity (Dell'Anno et al., 2023). Subsequent endotoxin translocation could amplify systemic inflammation (Tan P. et al., 2026; Wang Z. et al., 2026; Zhu et al., 2026), which, combined with stress responses (indicated by elevated cortisol and histamine), further compromises mammary efficiency (Ma et al., 2026; Tan J. et al., 2026). Furthermore, fecal microbiota exhibited more pronounced alterations than milk microbiota, strongly correlating with host inflammatory and metabolic traits. This indicates that gut microbial dysbiosis is a key covariate of the systemic inflammatory state rather than solely a downstream consequence. Taken together, laminitis is correlated with systemic inflammation, intestinal barrier dysfunction, and gut microbiota alterations, which collectively coincide with secondary impairments in mammary productivity.

An important observation in this study was the divergent responses of the milk and fecal microbiota. Contrary to the dense and metabolically active gastrointestinal ecosystem, the milk microbiota showed weak overall structural shifts and failed to generate a reliable predictive machine learning model (Sasidharan Pillai and Ashraf, 2026). This lack of a robust disease signature may be attributed to the fact that bovine milk represents a low-biomass microbial niche, making it highly susceptible to environmental noise and less reflective of consistent systemic states (Jyoti and Dey, 2025). In stark contrast, the fecal microbiota exhibited significant taxonomic shifts that robustly correlated with host systemic inflammation and barrier dysfunction. Furthermore, while direct metatranscriptomic measurements are lacking, the predicted genomic profiles of the fecal microbiota suggest potential functional adaptations to host stress conditions (Yang and Hammamieh, 2026). Collectively, these findings align with the previous results, demonstrating that the gut microbiota more accurately captures the systemic inflammatory responses associated with laminitis. Meanwhile, the true biological signal of the milk microbiome may either remain relatively stable or be obscured by low-biomass limitations and secondary physiological stressors (Wang Y. et al., 2026).

The observed microbial functional alterations are more plausibly interpreted as a secondary covariate of laminitis rather than a primary causal driver. Laminitis-associated pain and altered feeding behavior may disturb intestinal homeostasis, including nutrient availability and luminal pH (Lin et al., 2025). Within this altered host context, the predicted functional profiles of the gut microbiota likely reflect a genomic adaptation to environmental instability (Lozupone et al., 2012; Zaneveld et al., 2017). Specifically, the inferred enrichment of pathways related to transcription (K03088, K07090), transport, and redox metabolism (K02016, K00058) in laminitic cows suggests an enriched predicted genetic potential for stress-responsiveness and altered fermentation conditions (Elmhadi et al., 2022). Conversely, the microbiota of healthy cows exhibited a higher predicted genomic capacity for nutrient processing (K01262, K13038, K05349). It must be emphasized that these are computationally inferred potentials based on 16S rRNA data, not measured biological activities. Furthermore, these predicted microbial shifts coincided with elevated host LPS, DAO, and D-lactate levels, indicating compromised barrier integrity. Rather than directly driving systemic inflammation, this altered microbial genomic capacity likely co-occurs with barrier dysfunction, representing a coordinated, non-causal physiological response to the diseased host state.

Rather than reflecting isolated compositional shifts, the microbial patterns observed in this study are consistent with a broader physiological response to disease-associated stress (Lin et al., 2025). The coordinated correlations among gut microbial taxa, systemic inflammatory markers, and intestinal barrier indicators support the concept that these microbiota alterations represent a coordinated response to systemic host disturbances. Similar associations have been reported in other bovine metabolic disorders (Guo et al., 2021). where intestinal dysbiosis correlates with endotoxemia and systemic inflammation, which can secondarily impact distant organs such as the mammary gland (Yang et al., 2025). While our microbial co-abundance network analyses and machine learning models remain strictly exploratory due to the limited sample size, the inferred disruption of microbial interactions and predicted functional shifts align with this systemic perspective (Lin et al., 2025). By integrating host physiological traits with these exploratory microbial predictions, this study frames laminitis not merely as a localized hoof lesion, but as a complex disorder broadly correlated with systemic inflammation, secondary productivity impairments, and concurrent gut microbiota alterations.

In summary, laminitis is associated with coordinated alterations in production performance, systemic physiology, and microbiota dynamics. Beyond a localized hoof disorder, laminitis is correlated with broader host physiological disturbances characterized by impaired productivity, systemic inflammation, intestinal barrier dysfunction, and concurrent microbial dysbiosis. The more pronounced taxonomic shifts observed in the fecal compared with the milk microbiota highlight the gut as a sensitive covariate of disease-associated systemic disturbance. Together, these findings support a gut–systemic inflammatory link accompanied by secondary mammary productivity impairments. This perspective highlights potential biomarkers for disease monitoring, though extensive validation is required.

Several limitations of this study should be noted. First, the cross-sectional design precludes establishing causal relationships between laminitis and microbiota dysbiosis. Second, the small sample size limits the statistical power of our machine learning models, rendering them strictly exploratory. Finally, the PICRUSt-based functional profiles are estimations rather than direct measurements. Future longitudinal studies using multi-omics (e.g., shotgun metagenomics and metabolomics) in larger cohorts are warranted to validate these findings and elucidate causal mechanisms.

## 5 Conclusion

In conclusion, this pilot study demonstrates that laminitis in dairy cows is preliminarily associated with coordinated alterations in production performance, systemic physiology, and microbiota structure and function. Given the small cohort size, the current microbial network inferences and machine learning predictions are strictly exploratory in nature. Nevertheless, the integration of clinical traits, serum biomarkers, microbial profiles, and functional predictions proposes a hypothetical microbiota-associated gut–systemic interaction, accompanied by secondary mammary physiological dysfunction, during laminitis. These findings provide new insight into the systemic nature of laminitis and suggest that understanding microbiota-related mechanisms may offer potential strategies for disease monitoring.

## Data Availability

The dataset from this study has been uploaded to the National Center for Biotechnology Information (NCBI) database, with the accession number PRJNA943133.
