# Supplementary material for: Laminitis in Holstein dairy cows is associated with intestinal and mammary dysfunction, systemic inflammation, and microbiota dysregulation
Source: Front Microbiol. 2026 Jun 24;17:1879424. doi: 10.3389/fmicb.2026.1879424 (PMC13341666; doi:10.3389/fmicb.2026.1879424)
Supplement: Supplementary file 1 [file Supplementary_file_1.DOCX]

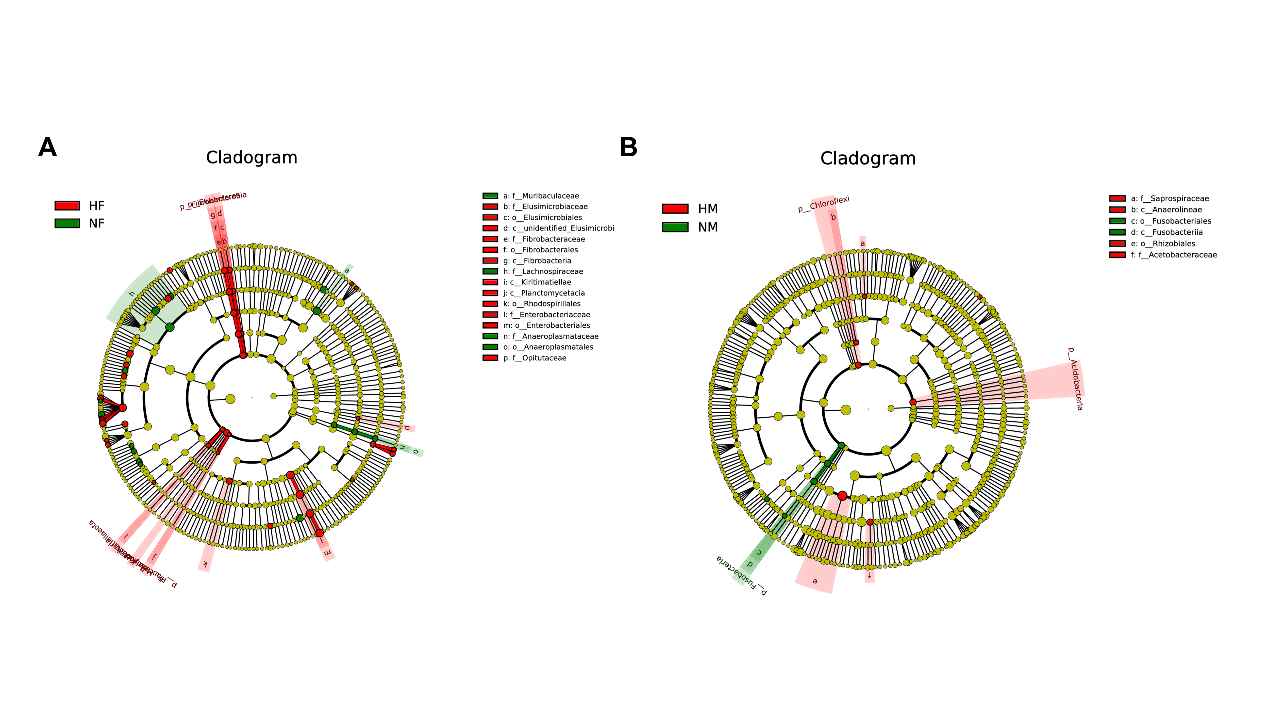


**Fig S1. Cladogram of differential microbial taxa identified by LEfSe analysis**

A: LEfSe lineage diagram of fecal samples samples from healthy cows and cows with laminitis.

B: LEfSe lineage diagram of milk samples from healthy cows and cows with laminitis.

Table S1. Ingredients and nutrient composition of the basal total mixed ration (TMR)

| Items | Content |
| --- | --- |
| Ingredients (% of dry matter) |  |
| Whole-plant corn silage | 35 |
| Alfalfa hay | 12 |
| Oat hay | 6 |
| Steam-flaked corn | 16.5 |
| Soybean meal | 11.5 |
| Corn DDGS | 5 |
| Sugar beet pulp | 6 |
| Cottonseed meal | 4 |
| Bypass fat supplement | 1.5 |
| Limestone | 0.8 |
| Sodium bicarbonate | 0.8 |
| Salt | 0.4 |
| Vitamin and mineral premix* | 0.5 |
| Nutrient composition (% of dry matter) |  |
| Net energy for lactation (NE_L, MJ/kg)** | 7.15 |
| Crude protein (CP) | 16.8 |
| Neutral detergent fiber (NDF) | 32.5 |
| Acid detergent fiber (ADF) | 20.2 |
| Starch | 24.5 |
| Ether extract (EE) | 4.6 |
| Ash | 7.2 |
| Calcium (Ca) | 0.95 |
| Phosphorus (P) | 0.42 |

Footnotes: > * The premix provided the following per kg of diet: vitamin A 8,000 IU, vitamin D3 2,000 IU, vitamin E 30 mg, Cu 15 mg, Fe 50 mg, Zn 50 mg, Mn 20 mg, I 0.5 mg, Se 0.3 mg, and Co 0.1 mg.

** NE_L (Net energy for lactation) was a calculated value based on the NRC (2001) model; other nutrient levels were measured values.

Table S2. Clinical characteristics and hoof lesion types of the laminitic dairy cows

| Cow ID | Primary Hoof Lesion Type | Affected Limb(s) | Lesion Severity |
| --- | --- | --- | --- |
| 01 | Sole hemorrhage | Right hind | Moderate |
| 02 | Sole ulcer | Left hind | Severe |
| 03 | White line disease | Right hind | Moderate |
| 04 | Sole ulcer | Both hind | Severe |
| 05 | White line disease | Left hind | Moderate |
